# Leveraging public health nurses for disaster risk communication in Fukushima City: a qualitative analysis of nurses' written records of parenting counseling and peer discussions

**DOI:** 10.1186/1472-6963-14-129

**Published:** 2014-03-19

**Authors:** Aya Goto, Rima E Rudd, Alden Y Lai, Kazuki Yoshida, Yuu Suzuki, Donald D Halstead, Hiromi Yoshida-Komiya, Michael R Reich

**Affiliations:** 1 Department of Public Health, Fukushima Medical University School of Medicine, Hikarigaoka 1, Fukushima-City, Fukushima 960-1295, Japan; 2 Takemi Program in International Health, Harvard School of Public Health (at time of manuscript writing), 665 Huntington Avenue, Boston, MA 02115, USA; 3 Department of Social and Behavioral Sciences, Harvard School of Public Health, 677 Huntington Avenue, Boston, MA 02115, USA; 4 Department of Health Communication, School of Public Health, The University of Tokyo (at time of manuscript writing), 7-3-1 Bunkyo-ku, Hongo, Tokyo 113-8654, Japan; 5 Office for Educational Programs, Harvard School of Public Health, 665 Huntington Avenue, Boston, MA 02115, USA; 6 Gender-Specific Medicine Center, Fukushima Medical University School of Medicine, Hikarigaoka 1, Fukushima-city, Fukushima 960-1295, Japan; 7 Department of Global Health and Population, Harvard School of Public Health, 665 Huntington Avenue, Boston, MA 02115, USA

**Keywords:** Public health nurses, Risk communication, Parenting, Radiation, Health communication, Fukushima nuclear accident, Japan

## Abstract

**Background:**

Local public health nurses (PHNs) have been recognized as the main health service providers in communities in Japan. The Fukushima nuclear disaster in 2011 has, however, created a major challenge for them in responding to mothers’ concerns. This was in part due to difficulties in assessing, understanding and communicating health risks on low-dose radiation exposure. In order to guide the development of risk communication plans, this study sought to investigate mothers’ primary concerns and possible solutions perceived by a core healthcare profession like the PHNs.

**Methods:**

A total of 150 records from parenting counseling sessions conducted between PHNs and mothers who have attended mandatory 18-month health checkups for their children at the Fukushima City Health and Welfare Center in 2010, 2011 (year of disaster) and 2012 were examined. Discussion notes of three peer discussions among PHNs organized in response to the nuclear disaster in 2012 and 2013 were also analyzed. All transcribed data were first subjected to text mining to list the words according to their frequencies and inter-relationships. The Steps Coding and Theorization method was then undertaken as a framework for qualitative analysis.

**Results:**

PHNs noted mothers to have considerable needs for information on radiation risks as they impact on decisions related to relocations, concerns for child safety, and experiences with interpersonal conflicts within the family owing to differing risk perceptions. PHNs identified themselves as the information channels in the community, recommended the building of their risk communication capacities to support residents in making well-informed decisions, and advocated for self-measurement of radiation levels to increase residents’ sense of control. PHNs also suggested a more standardized form of information dissemination and an expansion of community-based counseling services.

**Conclusions:**

Inadequate risk communication on radiation in the Fukushima nuclear incident has resulted in multiple repercussions for mothers in the community. Empowerment of local residents to assume more active roles in the understanding of their environment, increasing PHNs’ capacity in communication, and an expansion of health services such as counseling will together better address risk communication challenges in post-disaster recovery efforts.

## Background

Experience from past nuclear accidents shows that poor risk communication increases uncertainty and panic among the public [1], which has also been observed after the Fukushima nuclear disaster that occurred on March 11, 2011 following the Great East Japan Earthquake. The central government of Japan failed to inform the municipal governments of the occurrence and severity of the incident in a timely manner, leading to chaotic migrations among residents, and eventually causing excess mortality among vulnerable populations such as the institutionalized elderly [2,3]. Mothers of young children are among the most-affected in the Fukushima nuclear incident, as inconsistent information about radiation levels in breast milk posted by two different professional organizations (the Japanese Society of Obstetrics and Gynecology and the Japan Radiological Society) had further created high levels of confusion in terms of maintaining safety for their children [4].

This confusion around risk-related information continued into 2013 [5]. Two years after the disaster, the World Health Organization reported that in the two most affected locations of the Fukushima prefecture, the lifetime risk of thyroid cancer among girls exposed as infants had increased by up to 70%. However, the baseline risk is low and even a large relative increase only represents an absolute increase of as low as 0.005 in terms of life-time cumulated probability of developing the cancer [6]. The concept of risk is difficult to understand, even among health professionals, and its comprehension is even more complicated when a relative risk is high in spite of a low absolute risk [7]. This leads to major challenges for people, especially for families with small children in deciding on how to address the risks they are exposed to. In this case, Fukushima City is located about 70 km from the nuclear power plant, and the estimated additional lifetime risk of thyroid cancer is approximately half of that in the two locations mentioned above [6]. Nevertheless, the population of children under 5 years old in the city had declined by nearly 15% during the two years following the disaster.

Although the importance of empowering local residents via proper risk communication to allow them to make autonomous decisions in post-disaster recovery processes is recognized and recommended in Japan at the national level [2], no guidelines have been developed on how to plan or implement this at the community level. This has led to confusion among public health nurses (PHNs), who in Japan work in a public sector, provide a wide range of community health services, and are often the first point of contact with residents. The Japanese government has recognized the pivotal role they play, as PHNs are tasked with community assessment, health planning, service provision, networking and service evaluation at the municipal level. However, PHNs have voiced concerns about their safety and their insufficient level of knowledge to provide adequate services in the aftermath of the nuclear disaster [8]. In the year before the disaster, researchers from the National Mental Health Center in Japan had already revised the disaster mental health guidelines that were endorsed by mental health professionals. However, an agreement was not reached in defining the roles of PHNs in a disaster setting [9].

To develop proper maternal and child health strategies that can be adopted to support residents facing anxiety about radiation, regular meetings were held in Fukushima City in 2011 between a team of public health researchers from Fukushima Medical University (organized by the first author) and city PHNs in reaction to the disaster [10]. These meetings were expanded in the subsequent year to include three training workshops, aiming to provide PHNs with knowledge on the health effects of radiation exposure and create opportunities for information sharing among peer nurses. In addition, there were collaborations with PHNs to derive additional insight from their routine work. This included the analyses of child health checkup records written by PHNs, with particular attention to information from parenting counseling sessions conducted between PHNs and mothers, as they are one of the most affected population groups in the disaster. Mothers in Japan are required to report their pregnancies to a municipal office, and municipalities are mandated to provide health checkups for 18 and 36 months old children. PHNs are the main service providers of such checkups, as they assess children’s physical health while providing mothers with parenting counseling. As this community model of needs assessment and strategy planning by PHNs with technical support from a local university amidst an emergency situation is rare, both in the Fukushima context and scientific literature on disaster recovery, the understanding derived from this model is instrumental in alleviating existing post-disaster challenges in Japan and in future disaster occurrences.

The concerns of disaster-affected residents have to be carefully examined so that communication needs and strategies for improving risk communication in a community setting following a disaster can be appropriately delineated. This study focuses on mothers and PHNs (given their role in the Japanese community health setting) following the nuclear incident in Fukushima. Launching new systematic surveys in a post-disaster situation was not feasible. Concern with not overburdening either community members or professionals, information from existing healthcare services and disaster recovery efforts (i.e., mandated child health checkups and the afore-mentioned community model between the University and city PHNs) were capitalized upon in Fukushima City to undertake this line of academic inquiry.

**Figure 1:**
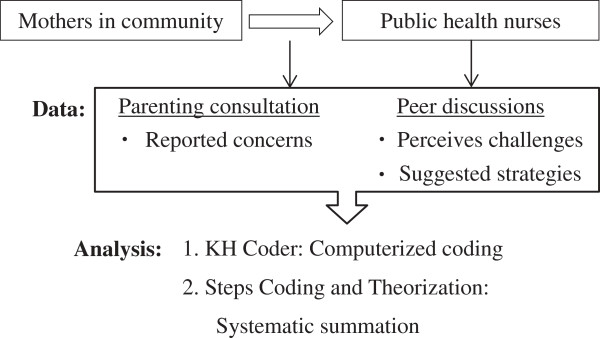
Framework for data analyses.

In this study, a qualitative approach has been adopted to look at existing records of parenting counseling to examine the residents’ concerns after the disaster with the nuclear incident, and PHNs’ peer discussions to delineate possible strategies that can be taken to alleviate these concerns (see Figure 1). The overarching aim is to elicit insights into steps that can be undertaken for risk communication strategies during disaster recovery efforts. Although health planning processes of PHNs have been investigated by previous research [11], little is known about the challenges they face in a crisis setting. The primary advantage of our study design is the possibility of documenting concerns within the community before and after the disaster, as a significant limitation in disaster studies is often the lack of baseline data [12]. Moreover, using two different sources of qualitative data have allowed us to explore issues from varying perspectives. This research will inform current and future efforts in disaster recovery, especially in terms of facilitating appropriate communication on topics of major concern in situations of disasters and crises.

## Methods

This was a qualitative analysis study utilizing two available data collected at the Fukushima City Health and Welfare Center and applying two analytical methods that are highly practical.

### Data collection

Data was drawn from parenting counseling records and from peer discussions among PHNs.

1. Parenting counseling records

Both quantitative and qualitative data on parenting counseling were collected from a series of cross-sectional studies consisting of mothers who attended their children’s 18-month health checkups at the Fukushima City Health and Welfare Center between April to May of 2010, 2011 and 2012. In Japan, mothers are required to report their pregnancies to a municipal office, and the municipal offices are in turn mandated to provide child health checkups. The attendance rate of the 18-month health checkups in Fukushima City for these three years was over 90%. It was one month after the disaster (i.e., April 2011) when regular health services including children’s health checkups resumed in the city. Based on sample size calculation for analyses of quantitative data (to detect expected associations), the study period was specified to be from April to May. The number of mothers enrolled in the checkups was 156 in 2010, 218 in 2011, and 131 in 2012. For each year, PHNs’ records from parenting counseling were scanned from the child health checkup files for the first consecutive 50 attendants and then transcribed into a database. Based on our previous pilot study, it was known that approximately 30% of mothers would voice concerns about effects of radiation in parenting counseling after the disaster [10]. The amount of qualitative data to be derived from each counseling session was also known. Coupling these insights, it was discerned with consensus among the authors involved in data collection that a sample size of 50 records of parenting counseling sessions per year will generate sufficient data for analyses.

2. Nurses’ peer discussions

We collected another set of data from PHNs attending three training workshops conducted in July and October 2012, and January 2013. These workshops were co-organized by the Gender-Specific Medicine Center and Department of Public Health of Fukushima Medical University School of Medicine, aiming to provide professional training and information sharing opportunities for PHNs in Fukushima City. The number of attendants was 20, 29 and 25 in each workshop, respectively. There were 39 PHNs working at the Fukushima City Health and Welfare Center at the time of study, and all except those assigned to service provision duties when the workshops took place attended. Each workshop consisted of a one-hour lecture by a university physician, followed by an hour of group discussion. The topics for each lecture were, in order of occurrence, women’s health, radiation, and thyroid cancer. In the group discussion, we handed each PHN three note sheets and asked them to write down their reflections after the lectures. All collected note sheets were then systematically transcribed into our database.

### Analysis plan

Both datasets were subjected to identical analysis methods (refer to Figure 1) - transcribed data were first analyzed by text mining using the KH Coder, a software program developed by K. Higuchi at Ritsumeikan University in Japan [13]. This text mining is a computerized process of extracting information from collected information, and has increasingly received attention among researchers as a way to improve the consistency of qualitative analysis, especially during the coding stage [14]. The KH coder has recently been used to analyze medical articles in academic journals and newspapers in Japanese [15]. The program segments sentences, lists frequently used words, and develops a hierarchical analysis diagram showing relationships among words with their corresponding frequencies.

We then focused on the top 20% of the words that were most frequently listed, and reviewed sentences that included these words to examine the full context in which the words were used. By referring to a diagram generated by the KH coder, we then categorized the words into major topics, eliminating common words that appeared in sentences across the different topics after categorization (e.g., “mother” and “think” in the parenting counseling data). We then calculated the proportion of cases that included each topic. A “case” refers to a mother in the parenting counseling data, or a note sheet from the PHNs’ workshop data. Since the KH Coder calculates word frequencies without analyzing the full context, some misclassifications were observed and manually eliminated when calculating this proportion of cases in each topic. This analysis procedure was repeated twice by the first author with a one-month interval in between to ensure consistency.

Second, cases related to specific topics of our interest (such as “disaster-related issues” among the parenting counseling data, and “action plans” among the workshop data) were analyzed by Steps Coding and Theorization (SCAT) [16]. SCAT was developed as an accessible qualitative data analysis method by T. Otani from Nagoya University in Japan. This method is appropriate for small-scale qualitative studies with a limited amount of qualitative data including answers to open-ended questions in surveys, and has been used by researchers in the fields of medical education and palliative care [17]. The analysis consists of two steps – first decontextualization to generate themes from sentences, followed by theorization to construct theories summarizing collected information. In the first decontextualization process, we extracted key words from original sentences, rephrased them by using professional terms, created themes, and then labeled each case. In the second recontexualization process, we developed a storyline from the emerging themes. The first and third authors independently performed the SCAT analysis of data extracted from each workshop, and the first author then compared and combined the results, with consensus achieved with the third author subsequently.

Compared with the classic grounded theory for qualitative analysis, which often requires a large amount of text data, the KH coder enhances consistency and reproducibility especially in data coding [14], and SCAT further enables systematic analysis of a small amount of text data [16]. We thus applied KH coder first to extract categories and then SCAT to deepen our interpretation of the categories that are most pertinent to our study’s objectives. One of this study’s aims is to test the usefulness of these methods in a community health practice setting.

### Ethical considerations

This study was conducted in collaboration with the Fukushima City Health and Welfare Center. All data were copied anonymously without any identifiers of families and PHNs, and conducted in accordance with Ethical Guidelines for Epidemiological Research issued by the Japanese Ministry of Education, Culture, Sports, Science and Technology, and the Japanese Ministry of Health, Labour and Welfare. Data collection on parenting counseling was submitted to the ethical review boards at the Fukushima Medical University and the city office, and reviews were waived. The data analysis plan of the PHNs’ group discussions was submitted to an IRB Review Specialist at the Harvard School of Public Health, but a review was also waived as this was categorized as a quality improvement study.

## Results

1. Parenting counseling

**Figure 2:**
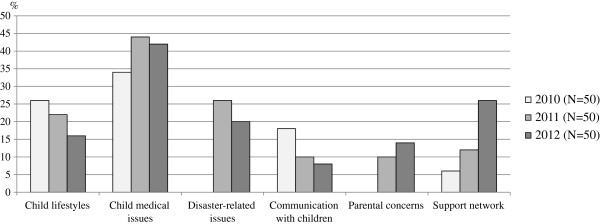
**Proportions of major topics: public health nurses’ records of parenting counseling.** Number of women (denominator) is 50 in each year.

Figure 2 shows trends found in parenting counseling topics categorized using the KH Coder. The proportions of “child lifestyle” and “communication with a child” decreased while those of “child medical issues”, “disaster-related issues,” “parental concerns,” and “support network” increased. The words included in each topic are listed in Table 1.

The cases that included words in the “disaster-related issues” topic—the records of 13 mothers in 2011 and 9 mothers in 2012—were analyzed by SCAT. In 2011, these mothers experienced relocation and changes in daily family routines. Mothers were worried about children’s radiation exposure from playing outside, and needed more information about radiation. These changes in daily life and anxiety toward radiation had negative psychological impacts. In 2012, mothers continued to relocate and voiced concerns about letting their children play outside. They started to ask questions about technical issues, including radiation measurement procedures, and raised concerns about differences in risk perception toward radiation with their spouses. PHNs recorded:

“[I was asked] What does it mean to measure a parent’s exposure level? Another city introduced a machine that a child can get into [and be measured directly].”

“The mother is worried about radiation and cannot let [child] play outside. [She worries about] her child licking the sand from her hands after falling down. The father says it is alright to play outside for a short time.”

2. Nurses’ peer discussions

**Figure 3:**
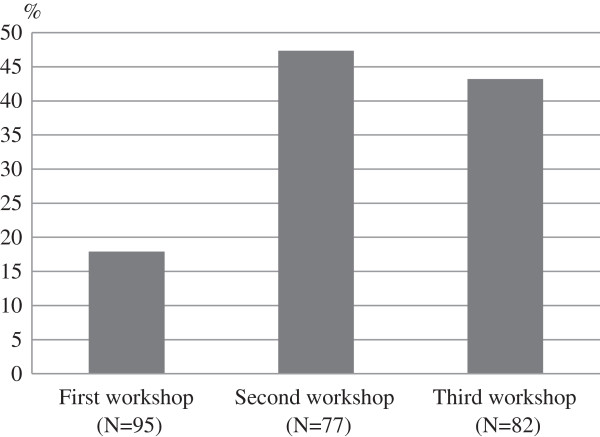
**Proportions of discussion notes including “action plans” words: public health nurses’ group discussions in workshops.** Denominator is the number of discussion notes in each workshop.

In the KH Coder analysis of the workshop data, we created two topics, “action plans” and “learned knowledge.” Since most of the notes included words categorized as “learned knowledge,” Figure 3 focuses on the proportion of notes including words categorized as “action plans.” We found that 18% of notes in the first workshop mentioned action plans, but this figure increased to 47% in the second workshop, and was 43% in the third workshop. Among words related to “action plans” as listed in Table 2, words (used as a noun or verb) that appeared across all three workshops were “inform” and “knowledge.”

The cases that included words in the “action plans” topic—the records of 17, 36, and 35 note sheets in three workshops, respectively—were analyzed by SCAT. PHNs identified their role as an information channel, and emphasized the importance of supporting residents to make autonomous decisions based on credible evidence. More specifically, they recommended measuring radiation levels as a useful tool for residents to have a clear understanding of their own environment. PHNs wrote:

“It is important for mothers to think by themselves based on accurate information. Public health nurses are their close advisors.”

“By measuring and calculating your own exposure level, anxiety and worries can be reduced.”

“[Use] radiation dosimeters. Calculate correctly.”

PHNs suggested the need to relay messages in a clearer manner and to have better community-based counseling service that addresses multiple but related concerns regarding parenting in the context of a disaster like Fukushima:

“Residents demand that we talk to them as the same disaster-affected residents. We must tell them what we think about what we have learned, not just what the government says.”

“It is important for public health nurses to learn and share knowledge.”

“[Mothers] need a place where they can ask every little question about their daily lives.”

In particular, there were strong concerns among PHNs that the purpose and results of thyroid cancer screening were difficult to understand, and that clearer, standardized explanations were needed. They further recognized that the matter was not only about improving access to information, but about providing a more careful follow-up system after the screening, taking its psychological impact into account:

“I understood how to read the screening results, but I am not confident in explaining them to residents.”

“[Once a thyroid cancer screening is introduced,] we will find cancer cases. It will look like the cancer is increasing. It becomes a problem of how to respond to people’s anxiety.”

**Table 1:** Words included in major topics: Public health nurses’ records of parenting counseling

**Major topics**	**Words in alphabetical order**
Child lifestyles	Banana, breast milk, crying at night, drink, eat, feed, irregular, meal, milk (two words), morning, nap, night, sleep (two words), vegetable
Child medical issues	Apply, bleed, brushing, curved, dry, ear, ear infection, eyes, feet, front teeth, height, immunization, legs, live (vaccine), medicine, nails, occlusion of teeth, pediatrics, prescription, prevention, polio, receive, run, skin, teeth, tooth decay, treat, vaccination, weight, words
Disaster-related issues	Daily life, earthquake, evacuation, Fukushima, nuclear, outside, play, radiation
Communication with children	Children, cry, dependent, dislike, elder child, hold
Parental concerns	Job, mind, overwhelmed, upset
Parenting network	Advice, child care, father, grandmother, nursery, parental home

**Table 2:** Words included in major topics: Public health nurses’ group discussions in workshops

**Workshops (Lecture topics)**	**Words categorized as “action plans” in alphabetical order**
First workshop (Women’s health)	Educate, important, inform, knowledge, necessary, promote
Second workshop (Radiation)	Calculate, close, confirm, correct, equip, execute, important, inform, information, knowledge, learn, mothers, necessary, play, prevention, public health nurses, understand
Third workshop (Thyroid cancer)	Adults, anxiety, children, correct, difficult, equip, explain, future, health, important, inform, information, issue, knowledge, manage, necessary, purpose, radiation, results, risk, safe, situation, think, worry

## Discussion

Given the uncertainty caused by inconsistent scientific information around radiation, the analyses of PHNs’ interactions with mothers and with their own peers have yielded insight into the challenges and corresponding strategies that PHNs face and can adopt for better communication of risks. This study provides community-based insights on the needs related to health communication and on the ways to improve risk communication following the Fukushima nuclear accident through the perspectives of PHNs, who live and work in, and interact regularly with the local community. This is the first study to delineate such information through a combination of data from PHNs and their interactions with mothers, and is thought to be instrumental to our general knowledge in disaster recovery interventions despite the context to be specific to the Fukushima Nuclear incident.

### Mothers’ concerns

Many disaster studies lack baseline data [12]; in contrast, our review of records from routine services of parenting counseling provides this critical baseline, and enabled us to document the changing trends of community concerns before and after the disaster. Many of the post disaster concerns from Fukushima are similar to those documented in other disaster settings [18,19]. The PHNs’ records of mothers’ reactions after the disaster were similar to those observed after the Chernobyl accident; parental anxiety at a subclinical level is expected according to previous research [18], and the mothers in our study have also raised similar concerns in the year of the Fukushima incident and in the following year. Another observation from the Chernobyl incident was the tendency for mothers to report somatic symptoms of their children despite no abnormalities in medical assessments [19], and our study has likewise observed an increase in consultations on children’s medical issues during health checkups in 2011.

Concerns about their children’s health further escalated, as evident from statements by mothers attending consultations in 2012 to question PHNs about technical information related to radiation and thyroid screening. Additional knowledge on radiation and its related health risks in PHNs is clearly warranted. Another finding from our study was how these mothers were also voicing conflicts of risk perceptions with their spouses. This resonates with findings from a recent study looking into risk communication after the Fukushima incident, which has shown a distinct higher proportion of mothers in Fukushima to have stronger levels of anxiety as opposed to fathers [20]. It is well known that risk perception differs between sexes, and perceived risk is generally higher among women than men [21]. In lieu of our findings, the strengthening of parenting support programs organized by the city currently is further recommended to address mothers’ persistent concerns on risk, and potential conflicts arising from differing risk perceptions within the family. This follows the Inter-Agency Standing Committee guidelines recommending strengthening of existing resources and capacities as one of core principles in mental health support in a disaster setting [22].

### Empowering mothers

PHNs, when considering mothers’ distinctive concerns, perceived themselves as information channels in the community and recognized the importance of empowering residents with advice so as to allow them to make well-informed decisions. The nurses proposed the provision and use of radiation dosimeters as an empowerment tool, and an expansion of the current scope of consultations to provide residents more opportunities to ask questions pertaining to daily life. The radiation dosimeters became widely sold commercially after the disaster, at a cost of about 30 USD. The availability and usage of radiation dosimeters is an important component for the regain of a sense of control of one’s surroundings, as demonstrated by an initiative supported by the European Commission ten years after the Chernobyl accident to successfully improve mothers’ sense of control by recommending the measuring of radiation levels in their living environments, and discussing protective measures that can be taken at home [23,24]. PHNs in Fukushima can therefore learn from these experiences and adopt similar activities in their local communities much earlier than the case of Chernobyl so as to better empower mothers and facilitate communication in radiation-related risks. Indeed, in line with recommendations by these post-Chernobyl studies [23,24], Fukushima City has already initiated experience-based learning sessions in which participating residents learn to measure and interpret their own radiation exposure level.

### Improving nurses’ health literacy skills

The PHNs also recognized their responsibilities and the need to improve their communication skills to better transfer scientific knowledge and information to the community. Previous research in health literacy has indicated that any information provided through parenting support and other city activities should be comprehensible to mothers and their family members [25]. Such access to information would allow residents to make informed lifestyle decisions. This might also relieve unnecessary distress in mothers and within the family. In times of crisis, high quality communication between lay people and healthcare workers is critical both in the relaying of clear and credible information and the engagement of intended audiences [26]. The National Action Plan to Improve Health Literacy has therefore been initiated in the US in 2010, with the goal of developing and disseminating health information that is accurate, accessible, and actionable [27]. Henceforth, increasing awareness of health literacy, a concept only introduced in Japan in the late 1990s [28], is an important step to further bolster quality communication between PHNs and community residents. Although no studies have been published on this front, Fukushima City and Fukushima Medical University are currently organizing and evaluating health literacy workshops for the PHNs.

### Expanding city health services

Going beyond the individual capacity-building of residents and PHNs, the nurses have also, in their peer discussions, suggested an organizational upgrading of the provision of city health services to improve their communication with the community. Specific suggestions included expanding the frequency of community-based counseling services, and standardizing the provision of information (highlighting the need for systematic information especially with regards to thyroid cancer screening). The importance of improving communication between the community and the local government office, in which PHNs work, is supported by a previous study [29]. In their examination of determinants of people’s trust toward government and non-government organizations, they found that an important factor to facilitate government-community relations was the degree of “openness” of an organization attained via the bidirectional communication. In general, a challenge among PHNs working in Japan’s hierarchical local government system is the difficulty of initiating new activities that have not been endorsed by the upper levels of the government [30]. The disaster has however, increased the PHN’s decision-making power in the local government system, thus new programs such as a mental health support program for mothers at the 18-month child health checkups, regular lectures on radiation-related topics for residents, and epidemiological assessments of child growth have been implemented since the Fukushima nuclear incident [10].

Although human resource and budget constraints exist, and the differing interests among stakeholders (such as upper boards of prefectural government, municipal office and universities) will need to be taken into account, continuous efforts to improve services that can enhance communication with the residents are required to address the social distrust caused in part by how the Japanese government has communicated with people immediately after the disaster [31].

### Study limitations and strengths

A major limitation of the present study was the limited amount of qualitative data extracted from the child health checkup files and workshop discussion notes. Each case contained only a few sentences that were often fragments. Another limitation was how samples were only collected within a specific time period of each year (between April and May). However, the qualitative analysis of both parenting counseling records and discussion notes from PHNs’ training workshops, although limited in amount and time-frame, can provide comprehensive information for PHNs and policy makers to understand community needs and to formulate appropriate strategies. Of note, a comparison between the present study and a previous study adopting a similar sampling strategy throughout a year in the sample city has indicated no differences in mothers and children’s basic characteristics [32].

On the other hand, a methodological strength of this paper was that it has demonstrated the usefulness of the combination of text mining and SCAT for the analysis of text data that are readily available and accessible via currently available healthcare services.

## Conclusion

The analyses of existing parenting counseling records and PHNs’ peer discussions have provided a deeper understanding of the challenges pertaining to risk communication that exist in the community and the possible strategies that PHNs can act on following a disaster in Japan. These challenges include the need for mothers to be considerably informed on radiation risks as they can have impact on their relocation decisions, child safety concerns, and interpersonal conflicts within the family due to differing risk perceptions. In addition, this study has shown how PHNs perceive themselves as information channels within the community; yet they acknowledge their lack of capacity to provide information and to communicate in a way that facilitates an optimal delivery of healthcare services in a disaster setting. Our study has therefore suggested a three-tiered strategy at the resident, healthcare provider and health system levels to alleviate these challenges of risk communication: empowerment of local residents to assume more active roles in the understanding of their environment and subsequently making informed decisions about preventive measures against radiation exposure, capacity-building of PHNs in health literacy skills so as to allow them to be sources of reliable communication channels, and the expansion of health services to enhance communications between PHNs and communities. As our findings have provided a premise for the initiation of capacity-building specific to health literacy in PHNs, the Fukushima Medical University has in collaboration with some local government offices started to conduct health literacy training sessions for the PHNs. It is anticipated that the recommendations from this research will inform future efforts in disaster recovery and the strengthening of current health and social care systems during times of crisis.

## Competing interests

The authors declare that they have no competing financial interests.

## Authors’ contributions

AG designed the study, analyzed data and wrote the manuscript. RER and AYL contributed to data analysis, interpretation and manuscript writing. KY contributed to data collection and data management. YS conceived of the study and collected data. DDH contributed to manuscript planning and writing. HK supervised the workshop and contributed to manuscript writing. MRR contributed to data interpretation and manuscript writing. All authors read and approved the final manuscript.

## Pre-publication history

The pre-publication history for this paper can be accessed here:

http://www.biomedcentral.com/1472-6963/14/129/prepub
